# Determinants of antenatal care, institutional delivery and postnatal care services utilization in Nigeria

**DOI:** 10.11604/pamj.2015.21.321.6527

**Published:** 2015-08-31

**Authors:** Tukur Dahiru, Oche Mansur Oche

**Affiliations:** 1 Department of Community Medicine, Ahmadu Bello University, Zaria, Kaduna State, Nigeria; 2 Department of Community Health, Usmanu Danfodio University, Sokoto, Sokoto State, Nigeria

**Keywords:** ANC, institutional delivery, postnatal, Nigeria, DHS, 2013

## Abstract

**Introduction:**

Utilization of antenatal care, institutional delivery and postnatal care services in Nigeria are poor even by african average.

**Methods:**

We analysed the 2013 Nigeria DHS to determine factors associated with utilization of these health MCH indicators by employing both bivariate and multivariate logistic regressions.

**Results:**

Overall, 54% of women had at least four ANC visits, 37% delivered in health facility and 29% of new born had postnatal care within two of births. Factors that consistently predict the utilization of the three MCH services are maternal and husband's level education, place of residence, wealth level and parity. Antenatal care strongly predicts both health facility delivery (OR = 2.16, 95%CI: 1.99-2.34) and postnatal care utilization (OR = 4.67, 95%CI: 3.95-5.54); while health facility delivery equally predicting postnatal care (OR = 2.84, 95%CI: 2.20-2.80).

**Conclusion:**

Improving utilization of these three MCH indicators will require targeting women in the rural areas and those with low level of education as well as creating demand for health facility delivery. Improving ANC use by making it available and accessible will have a multiplier effect of improving facility delivery which will lead to improved postnatal care utilization.

## Introduction

Recent estimates show that the overall ante-natal care (ANC) coverage in Nigeria stood at 61% which is an abysmal three percentage points increase from 58% a decade ago; 36% of deliveries were delivered in a health facility while only 14% of newborns received postnatal care within two months of delivery [1]. The ANC coverage of 61% falls short of the recommended 90% of ANC coverage required to reduce most deaths among mothers and their newborn [2]. Additionally, this national average conceals major variations between rural and urban areas as well as between states and geopolitical zones within the country. For instance, the rural and urban ANC coverages are 47% and 86% respectively; the North West zone has the least of 41% while the South East has the highest coverage of 91%. Institutional delivery show similar regional and rural/urban disparity where it is highest in urban areas (63%) than in rural areas (23%); highest in South West zone (76%) and lowest in North West (12%); the consequence of these differentials of coverage in ANC is that maternal and child health mortality remains high (as well as other indicators of maternal and child health) at national level despite the co-existence of high national coverage in ANC [1]. Furthermore, despite high coverage of ANC, Nigeria still remains a major contributor of under-five mortality, contributing about 13%, 9.4% and 14% of global under-five, neonatal and maternal deaths respectively [3, 4]. Expectedly the maternal mortality, neonatal, post-neonatal, infant, child and under-5 mortality rates remain high at 576 maternal deaths per 100000 live births; 37, 31, 69, 64 and 128 per 1000 live births respectively. These figures show a reduction of between 20% and 31% in the past decade but not enough to achieve MDGs 4 and 5 [1].

In the continuum of maternal health care, antenatal care, institutional/skilled attendance at delivery and postnatal care are important milestones required to achieve optimum maternal and child health. These elements of care are expected to be provided as a continuum of care in order to impact optimum benefit and the provision of these elements of care in a comprehensive and continuum pattern of care during pregnancy, child birth and postpartum period has been argued to reduce maternal and child (neonatal) death [2]. Firstly, antenatal care which is entry point for maternal and child care service utilization has the capability to reduce both maternal and neonatal mortality by detecting at-risk pregnancy and managing the risk associated. It also has additional secondary benefit of providing a platform for interaction between the medical personnel and the pregnant woman during which relevant information and education concerning the health of the mother and her unborn child is passed as well as screening for infections such as syphilis and HIV and other abnormalities and complications. Antenatal care affords the medical personnel the opportunity to detect and treat symptomless ailments such high blood pressure and pregnancy-induced diabetes and facilitates informed decision-making by the pregnant woman such as seeking skilled attendance at delivery and delivery in health care facility. All these interventions received by the pregnant woman during ANC have the potential to improve the survival chance of herself and her newborn [4–7]. Additionally, an extended benefit of ANC is that women who utilized ANC are more likely to utilize institutional/skilled delivery [8]. The second element of care, institutional delivery/skilled attendance at delivery allows provision of intervention to detect risk around labour and childbirth during which interventions can maximally be provided by skilled medical personnel at health facilities [9]. The third element of care, postnatal care has been argued that promoting the utilization of ANC and institutional delivery/skilled attendance at delivery alone is not enough to improve maternal and child health and that postnatal care has to be provided to sustain the reduction in neonatal mortality [10, 11]. Furthermore, more than two-thirds of neonatal deaths occur within the first seven days of life and over half of these taking place in the first 24hours of life; implying that the first 24 hours of life are critical in newborn survival. However, in spite of its potential role in reducing newborn and maternal deaths, postnatal care has been one of the elements of newborn care that is poorly provided and poorly utilized; only around 14% of all newborns had post natal care within the first two days of delivery in Nigeria [1].

However, despite the benefits derived from utilization of focused antenatal care, institutional delivery and postnatal care in terms of reducing maternal and neonatal mortality, Nigeria does not seem to be making progress in terms of these services. While there are several studies documenting factors related to utilization of antenatal care and institutional delivery/skilled attendance at delivery in Nigeria [12–25] studies exploring utilization of postnatal care are scarce and fragmentary despite its role promoting maternal and neonatal health. Perhaps the available literature on utilization of postnatal care in Nigeria is that by Ononokpono [26]; this paucity of data on utilization of postnatal care has also been demonstrated in India where only two studies have been identified [10]. The aim of this study was to investigate the potential factors related to use/non-use of antenatal care, institutional delivery and postnatal care with the ultimate goal of providing relevant policy and programmatic recommendations as we prepare for the post-2015 agenda.

## Methods

The study utilized the 2013 Nigeria Demographic and Health Survey (NDHS) data sets available on the public domain through the Measure DHS website (www.measuredhs.com) [27]. The survey process went through the required ethical clearance procedures; however permission to use the dataset was granted by Measure DHS. The survey was cross-sectional descriptive collecting information from 38,945 eligible women and 17, 359 men all aged 15-49 years. The survey was conducted by the National Population Commission (NPC) in collaboration with ICF Macro, Calverton, MD, USA. The 2013 Nigeria DHS was the fifth in the series that began in 1 > 990 and it used a nationally-representative sample of women and men involving a stratified three-stage cluster sampling design. Administratively, Nigeria is made up of 36 states and a Federal Capital Territory (FCT); in each of the states and FCT there are local government areas (LGAs) which are the smallest administrative units recognized by the constitution. Broadly, the country is divided into urban and rural areas based on certain pre-defined criteria by the NPC for the purpose of census and large surveys like the DHS. The 2013 Nigeria DHS utilized the 2006 census enumeration area (EAs) to prepare the sampling frame from which sample of clusters, households and eligible respondents were selected. The EAs constituted the clusters of which there are 904; 372 in urban areas and 532 in rural areas selected for the purpose of this survey. From these 904 clusters, a representative sample of 40,680 household was selected ensuring a minimum sample of 45 households per cluster. All women aged 15-49years present, either permanent resident or a visitor in the household the night before the survey were eligible for the interview. Further, in a subsample of half of the households, all men aged 15-49years present either as permanent residents or visitors were eligible for the interview. The survey used basically three types of structured questionnaires on the respondents: the Household Questionnaire, Woman's Questionnaire and Man's Questionnaire. The Household Questionnaire listed all usual members and visitors to the selected households; additional basic information collected was on the characteristics of each person listed, including age, sex, marital status, education, and relationship to the head of the household. The Woman's Questionnaire collected information on background characteristics, reproductive history and childhood mortality, family planning methods, fertility preferences, antenatal, delivery, and postnatal care and host of other health issues relating to specific diseases and disease-prevention programmes/interventions. The Man's Questionnaire is similar to the Woman's Questionnaire except that it is shorter. At the end of the exercise, 99.0%, 97.6% and 95.2% response rates were recorded for the households, women's and men's responses [1]. Information's collected from these questionnaires were transformed into Stata data files for statistical analysis. For the purpose of this study, the birth record data file was used since it recorded antenatal care utilization by women who were pregnant within the five years period prior to the survey.

### Study variables

The outcome/dependent variables for this investigation are three. The first is whether or not a woman had at least four antenatal care visits during her most recent pregnancy in the five year period preceding the survey. The World Health Organization has recommended that pregnant women should have at least four ANC visits to maximize the benefits of ANC services especially if she is found not have any associated ill-health or condition that can jeopardize her health and that of her fetus/newborn; those with any associated risk would be required to make more frequent visits to receive adequate medical attention [28, 29]. This variable is dichotomous taking the value of ‘1’ if the woman has had at least four skilled ANC visits or ‘0’ if she did not have. In this model of focused or basic ANC, a skilled provider is defined as a doctor, a nurse or midwife, or auxiliary nurse or midwife. The second outcome variable is place of delivery which is also a dichotomous variable taking the value of ‘1’ if the woman delivered in health facility (both public and private are included here) and ‘0’ if she did not deliver in a health facility (that is either at home or any other places outside of health facility); no distinction here is made about skilled or non-skilled attendance at delivery and it is assumed that those who delivered at health facility had skilled attendance. Research has shown that skilled attendance at home does not have the potential to reduce adverse pregnancy outcomes such as maternal and neonatal deaths [30, 31]. The third outcome variable which is postnatal care is also dichotomous taking values of ‘0’ if the newborn did not received postnatal care and ‘1'if the new born received postnatal care within two months of delivery. Again, no distinction is made between skilled and non-skilled postnatal care and the postnatal care is restricted to newborns and not the mothers. These outcome variables are examined against all the confounding/controlling variables/covariates: sociodemographic and socio-economic factors of the woman. Table 1 provides a detailed description of all the variables used and their categorization.

**Table 1:** Definitions and categorization of variables used in the determinants of ANC and institutional delivery in Nigeria DHS 2013

Variable	Coding
Antenatal care	Attended at least four ANC visits during last pregnancy (Yes = 1;No = 0)
Place of delivery	Delivered in health facility during last delivery (HF = 1;Home = 0)
Post-natal care	New born received postnatal care within two months (Yes = 1; No = 0)
Maternal age	Maternal age at interview in groups of 5years from 15-19years (1-7)
Geopolitical zone	Geopolitical zone of residence; six zones coded 1-6
Place of residence	Place of residence coded 1-2; (rural = 2, urban = 1)
Maternal educational attainment	Highest educational level(none = 0; primary = 1; secondary = 2; higher = 3)
Husband's educational attainment	Highest educational level(none = 0; primary = 1; secondary = 2; tertiary = 3)
Wealth index	Household wealth index (poor = 1;middle = 2;rich = 3
Religion	Religious affiliation of mother (Christian/Catholic = 1; Islam = 2; Traditional/other = 3
Parity	Number of children given birth (one = 1;2-4 = 2;5 + = 3)
Cowives	Number of other wives (none = 0;1-3 = 1;4 + = 2)
Sex of household head	Sex of household head (male = 1; female = 2)
Pregnancy desire	Mother's desire for baby (wanted then = 1; wanted later = 2; wanted no more = 3)
Decision maker in household	The person making decision on health issues of the mother (Husband alone = 1; Woman and husband = 2; Woman alone = 3)
Health insurance	If the woman has health insurance coverage (Yes = 1; No = 0)

### Statistical analyses

First, descriptive statistics related to utilization of ANC, place of delivery and postnatal care were generated by means of frequency table as shown in Table 2. Crude odds ratios were generated by means univariate analyses to determine the odds of utilization of focused ANC, institutional delivery and postnatal care with variables enumerated in Table 1. Multivariate logistic regression analysis was used to determine the association between the three outcome variables and the explanatory/independent variables; all the independent variables used in univariate analyses were also used in multivariate since all were significantly related to the three outcome variables at 5% (i.e. p < 0.05) during univariate analyses. All the statistical analyses were conducted using Stata v12.0 (StataCorp, College Station, TX, USA) [32]. Multivariate binary logistic regressions were fitted using Stata complex survey command svy to adjust for the complex sampling design. Additionally, all output generated in the univariate and multivariate analyses were weighted with the appropriate sampling weights as per the DHS sampling scheme. However, before running the multivariate logistic analysis, backward stepwise elimination process was conducted to eliminate highly correlated variables before they are entered in the final model and only potential factors with p < 0.20 were used in the final model. Factors that showed collinearity were not included in the final model.

**Table 2:** Percent distribution of ANC and institutional delivery by women's characteristics, Nigeria DHS 2013

Variable	ANC visits (%)		Place of delivery (%)		Postnatal care (%)	
	<4	>4	Home	Health facility	No	Yes
**Age of mother**						
15-24	53.3	30.0	68.4	31.6	74.8	25.2
25-34	43.1	15.0	60.3	39.7	69.3	30.7
35 +	43.9	4.7	62.2	37.8	70.8	28.9
**Geopolitical zone**						
North Central	42.1	57.9	46.4	53.6	65.4	34.6
North East	58.8	41.2	79.1	20.9	75.8	24.2
North West	68.5	31.5	89.5	10.5	86.6	13.4
South East	12.7	87.3	20.5	79.5	69.0	31.0
South South	29.2	70.8	55.2	44.8	65.5	34.5
South West	9.7	90.3	25.2	74.8	41.9	58.1
**Place of residence**						
Urban	21.3	78.7	35.5	64.5	55.7	44.3
Rural	59.7	40.3	76.2	23.8	79.0	21.0
**Mother's level of education**						
No formal education	71.0	29.0	87.9	12.1	87.0	13.0
Primary	35.7	64.3	59.0	41.0	67.7	32.3
Secondary +	19.5	80.5	29.4	70.6	52.3	47.7
**Husband's level of education**						
No formal education	77.5	22.5	89.2	10.8	88.1	11.9
Primary	54.7	45.3	61.7	38.3	71.7	28.3
Secondary +	33.4	66.6	40.2	59.8	57.0	43.0
**Wealth index**						
Poor	72.1	27.9	87.4	12.6	86.8	13.2
Middle	40.0	60.0	60.7	39.3	70.5	29.5
Rich	16.1	83.9	31.1	68.9	52.0	48.0
**Religion**						
Christianity	23.8	76.2	40.1	59.9	59.7	40.3
Islam	59.6	40.4	78.2	21.8	79.1	20.9
Traditional/other	63.5	36.5	79.0	21.0	85.6	14.4
**Parity**						
One	41.5	58.5	50.0	50.0	65.4	34.6
2-4	44.5	55.5	57.3	42.7	68.6	31.4
5 +	55.4	44.6	72.7	27.3	76.8	23.2
**Sex of household head**						
Male	48.4	51.6	64.8	35.2	72.2	27.8
Female	26.1	73.9	45.6	54.4	62.6	37.4
**Pregnancy desire**						
Then	49.8	50.2	64.0	36.0	72.6	27.4
Later	33.0	67.0	51.3	48.7	58.7	41.3
No more	29.2	70.8	45.5	45.5	58.5	41.5
**Place of delivery**						
Home	65.9	34.1	na	na	89.0	11.0
Facility	13.1	86.9	na	na	52.0	48.0
**ANC visits**						
<4	na	na	76.3	23.7	95.8	11.0
4 +	na	na	40.3	59.7	61.0	48.0
**Health Insurance coverage**						
No	47.7	52.3	63.5	36.5	71.7	28.3
Yes	8.3	91.7	18.0	82.0	38.7	61.3
**Decision maker**						
Husband alone	26.1	73.9	37.6	62.4	63.2	36.8
Woman plus husband	29.6	70.4	43.2	56.8	58.6	41.4
Woman alone	58.5	41.5	75.0	25.0	78.9	21.1
**Total**	**46.0**	**54.0**	**63.7**	**37.3**	**71.1**	**28.9**

## Results

Table 2 shows the percent distribution of women with four or more antenatal care services, those delivering at home/health facilities in their recent pregnancy/birth and newborns who received post natal care. Overall, about 54% of women had at least four ANC visits during their last pregnancies five year period before the survey; 37.3% delivered in a health facility and 28.9% of newborn babies received postnatal care within two months of delivery. Regarding ANC utilization, women in the younger age group of 15-24year were more likely to have four ANC visits compared to women in the other age groups. Women in the southern part of the country utilized ANC services better compared to those in the northern of Nigeria; residing in urban area means more ANC visits than residing in rural areas (79% against 40%). Increasing level of woman's education as well as husband's level of education increases linearly with use of ANC; 81% of women with secondary level of education and above and 67% of women whose husbands had secondary level of education and more utilized at least four ANC visits compared to only 29% and 23% in each case without formal education. About 84% of women in the rich wealth bracket had at least four ANC visits while only 28% of women in the poor household have had four ANC visits. Women whose religious affiliation is Christianity have higher utilization rate of ANC than women of other religions. Women in households where the head is a female had more utilization rate (74%) than households with males (52%) as the heads. Women who wanted no more pregnancy (71%) where better users of ANC than those who wanted it then (50%) or those who wanted it later (67%). In retrospect, women who eventually delivered in health facility (87%) utilized ANC more frequently than those who did not (34%). Also having health insurance coverage increased the utilization (92%). Women who make decision together with their partners (70%) are marginally better utilizers of ANC than those women whom their husbands made the decision alone for health care service utilization (74%). With regards to institutional delivery, the results indicate that overall, 37% of deliveries occurred in health facilities increasing from 35% five years ago. Women in the middle age group of 25-34, living in the South East and South West geopolitical zone, as well as residing in urban area have more propensities to use health facilities for delivery. Women as well as women whose husbands have secondary education and more and living in the richest wealth index households utilized institutional delivery services more than those in the poorest or those with no formal educational or whose husbands have no formal education. Women who had at least four ANC visits were more likely to deliver in health facilities (56% of women with at least four ANC visits delivered in health facilities compared to 40% with less than four ANC visits). Women belonging to Christian faith (60%) utilized more of health facilities for deliveries than those in the other two religions. Also women with parity one (50%) utilized facilities for delivery more compared with those with higher parities. We added two additional variables: health insurance coverage and dimension of woman's autonomy with regard to decision making on health issues.

According to the 2013 Nigeria DHS, about 2.1% of respondents have some sort of health insurance coverage and having insurance coverage seemed to positively influence both the use of ANC and health facility delivery as opposed to non-enrollment (92% as against 52% for ANC use and 82% as against 37% for health facility delivery). If the woman is the sole decision maker for her health, she is less likely to deliver in health facility (25%) compared to if the husband is the sole decision maker (62%) or she makes the decision jointly with the husband (57%). Postnatal care utilization by newborns was assessed within two months of delivery and the results are shown in the last two columns of Table 2. Overall, about 29% of newborns had postnatal care within two months of their births; 14% taking place in respondent's home and 86% in health facilities. Age of woman does not seem to influence utilization of postnatal care for newborn; utilization rate by age group of women is tightly clustering between 25% and 31%. Expectedly, utilization rates are higher in Southern (41%) geopolitical zones than Northern zones (24%); higher in urban (44%) than in rural (21%) and linearly related with both maternal and husband's levels of education. Postnatal care utilization is also linearly related with household wealth index; those in the rich wealth bracket (48%) have more than three times the rate of those in the poor wealth bracket (13%). Again, those belong in the Christian faith (40%) have higher rate of utilization of postnatal care than those in the other two faiths. There is wide margin of utilization between women of parity one (35%) and those with parity five and more (23%). Likewise, the sex of the household head was women in female-headed households have higher utilization rate (37%) than those in male-headed households (28%). Women whom wanted their pregnancy then (27%) utilized postnatal care less than those whom wanted it later (41%) or wanted no more (42%). Facility delivery seems to facilitate utilization of postnatal care; about half (48%) of those who delivered in a health facility accessed postnatal care compared to only 11% among home delivery. Health insurance also plays to facilitate use of postnatal more as obtained with ANC and facility delivery.

### Univariate and multivariate analyses

The results of the univariate logistic regressions are shown in Table 3. Again, it shows similar semblance to the results obtained in Table 2. Generally, all the sociodemographic variables are significantly related to use of ANC utilization. Age wise, those older than 25years are much less likely to utilized ANC than those who are younger (OR = 0.42, 95%CI: 0.39-0.45). Residing in any of the other five geopolitical zones of Nigeria (besides South West) makes it less likely for the women to utilize ANC; women in these five zones have between 33% and 77% odds of utilizing ANC compared to those in South West. Rural location confers some disadvantage in use of ANC where they have odds of only 0.47 (95%CI: 0.45-0.49) of utilizing ANC compared to their urban counterparts. Both maternal and husband's levels of education showed increased odds of utilization with increasing educational attainments. Household wealth level has a similar pattern of increasing utilization by increasing household wealth level, rich household are 3.42 times the odds to utilize ANC than the poor households (95%CI: 3.25-3.61). Other covariates that independently increased the odds of ANC utilization are: female-headed households (OR = 1.15, 95%CI: 1.08-1.23), status of pregnancy wantedness, health insurance coverage (OR = 1.92, 95%CI: 1.66-2.22) and the status of the decision maker in the household. Those covariates that reduced the odds of ANC utilization are being employed (OR = 0.91, 95%CI: 0.87-0.96) and a high parity of two and more (OR = 0.16, 95%CI: 0.15-0.17). In terms of place of delivery, covariates that had independent significant influence on utilization of health facility for birthing included age of mother, maternal as well as husband's level of education, maternal employment status, household wealth level, sex of household head, pregnancy wantedness, use of at least four ANC visits, health insurance coverage and person deciding on utilizing of health care.

**Table 3:** Univariate analyses of determinants of antenatal care, place of delivery and postnatal care, 2013 Nigeria DHS

Variable	Antenatal care			Place of delivery			Postnatal care		
	OR	[95% CI]	P	OR	95% CI	P	OR	95% CI	P
**Age of women**									
15-24	1.00			1.00			1.00		
25-34	0.42	[0.11-0.13]	<0.001	1.46	[1.37-1.57]	<0.001	1.52	[1.38-1.68]	<0.001
35 +	0.12	[0.39-0.45]	<0.001	1.31	[1.21-1.42]	<0.001	1.37	[1.23-1.53]	<0.001
**Geopolitical zone**									
South West	1.00			1.00			1.00		
North Central	0.68	[0.63-0.73]	<0.001	0.28	[0.25-0.32]	<0.001	0.25	[0.22-0.28]	<0.001
North East	0.42	[0.39-0.46]	<0.001	0.08	[0.07-0.09]	<0.001	0.15	[0.13-0.17]	<0.001
North West	0.33	[0.30-0.35]	<0.001	0.04	[0.04-0.05]	<0.001	0.11	[0.09-0.12]	<0.001
South East	0.77	[0.71-0.84]	<0.001	1.29	[1.12-1.47]	<0.001	0.34	[0.29-0.40]	<0.001
South South	0.67	[0.62-0.73]	<0.001	0.33	[0.30-0.38]	<0.001	0.39	[0.33-0.45]	<0.001
**Place of residence**									
Urban	1.00			1.00			1.00		
Rural	0.47	[0.45-0.49]	<0.001	0.17	[0.16-0.18]	<0.001	0.23	[0.21-0.25]	<0.001
**Women's level of education**	1.00	1.00	1.00	1.00	1.00	1.00	1.00	1.00	1.00
No formal education	1.00			1.00			1.00		
Primary	2.05	[1.93-2.18]	<0.001	5.75	[5.28-6.25]	<0.001	4.43	[3.93-5.00]	<0.001
Secondary and above	4.49	[4.26-4.73]	<0.001	19.80	[18.32-21.41]	<0.001	10.48	[9.44-11.63]	<0.001
**Women's working status**									
No	1.00			1.00			1.00		
Yes	0.91	[0.87-0.96]	<0.001	1.81	[1.70-1.73]	<0.001	1.88	[1.72-2.06]	<0.001
**Husband's level of education**									
No formal education	1.00			1.00			1.00		
Primary	2.32	[2.17-2.48]	<0.001	5.60	[5.10-6.14]	<0.001	1.00	[3.68-4.83]	<0.001
Secondary and above	4.13	[3.91-4.37]	<0.001	13.80	[12.72-14.96]	<0.001	4.22	[8.25-10.37]	<0.001
**Wealth index**									
Poor	1.00			1.00			1.00		
Middle	2.17	[2.04-2.32]	<0.001	4.63	[4.25-5.04]	<0.001	3.51	[3.09-3.98]	<0.001
Rich	3.42	[3.25-3.61]	<0.001	16.81	[15.57-18.15]	<0.001	10.81	[9.73-12.02]	<0.001
**Religion**									
Traditional/other	1.00			1.00			1.00		
Christianity	3.18	[2.40-4.20]	<0.001	5.95	[4.22-8.38]	<0.001	4.76	[2.64-8.56]	<0.001
Islam	1.17	[1.30-2.27]	<0.001	0.88	[0.63-1.24]	0.469	1.36	[0.76-2.45]	0.300
**ANC use (at least 4visits)**									
No	na	na	na	1.00			1.00		
Yes	na	na	na	4.61	[4.34-4.88]	<0.001	16.15	[14.02-18.60]	<0.001
**Place of delivery**									
Home	na	na	na	na	Na	na	1.00		
Health facility	na	na	na	na	Na	na	8.32	[7.62-9.09]	<0.000

In terms of geopolitical zone, only residing in the South East (OR = 1.29, 95%CI: 1.12-1.47) increased the odds of delivering in health facility compared to residing in South West; residing in all other zones is associated with a decreased odds of delivering in a health facility. An important finding here is the relationship between obtaining at least four ANC visits and subsequent facility delivery; ANC utilization increased the odds of facility delivery by more than four times (OR = 4.61, 95%CI: 4.34-4.88). In terms of postnatal care utilization, it is clear from Table 3 that age of mother, maternal and husband's level of education, maternal employment, household wealth index, religion, sex of household head, pregnancy wantedness, utilization of ANC, health insurance coverage, decision maker in the household and place of delivery all significantly and consistently increased the odds of postnatal care for the newborns. Residing in any other geopolitical zone besides South West and in rural areas and being of parity two and more significantly reduced the odds of accessing postnatal care. The results of multivariate analysis are shown inTable 4. Consistent statistically significant relationship is seen between ANC and maternal age, place of residence, maternal and husband's level of education, household wealth level, religion and working status of woman. Sex of household head and pregnancy wantedness status also significantly increases the odds of utilization of ANC. Health insurance coverage, parity and person deciding on health issues of the mother are not significant determinants of ANC utilization (results not shown here). Residing in North West (OR = 0.83, 95%CI: 0.73-0.95), South East (OR = 0.80, 95%CI: 0.70-0.92), South South (OR = 0.62, 95%CI: 0.55-0.71) geopolitical zones all reduced the odds of ANC utilization. Likewise residing in rural areas (OR = 0.76, 95%CI: 0.709-0.82) and having parity of two or more (OR = 0.22, 95%CI: 0.20-0.25). Equally, covariates predicting utilization of ANC are similarly predicting facility delivery. Place of residence, maternal and husband's level of education, household wealth level and utilization of at least four ANC visits are the consistent and uniform predictors of facility delivery. Inconsistent predictors are maternal age, geopolitical zone of residence and religion. Non-predictors of facility delivery are maternal employment (OR = 1.03, 95%CI: 0.94-1.13), sex of household head (OR = 1.09, 95%CI: 0.95-1.26) and pregnancy wantedness. Covariates for postnatal care utilization included: maternal age, geopolitical zone, place of residence, maternal education, working status of mother, educational level of husband, household wealth level, parity, pregnancy wantedness, utilization of ANC and the decision maker in the household. Non-significant determinants of postnatal care utilization included sex of household head, health insurance coverage and religious affiliation.

**Table 4:** Adjusted odd ratios of the determinants of antenatal care, place of delivery and postnatal care 2013 Nigeria DHS

Variable	Antenatal care			Place of delivery			Postnatal care		
	OR	[95% CI]	P	OR	95% CI	P	OR	95% CI	P
**Age of women**									
15-24	1.00			1.00			1.00		
25-34	1.37	[1.25-1.50]	<0.001	1.03	[0.92-1.15]	0.582	1.18	[1.02-1.36]	0.023
35 +	2.15	[1.92-2.42]	<0.001	1.22	[1.06-1.40]	0.007	1.30	[1.09-1.56]	0.004
**Geopolitical zone**									
South West	1.00			1.00			1.00		
North Central	1.05	[0.93-1.19]	0.430	0.77	[0.67-0.89]	<0.001	0.57	[0.49-0.67]	<0.001
North East	1.01	[0.88-1.16]	0.874	0.39	[0.33-0.45]	<0.001	0.88	[0.73-1.06]	0.184
North West	0.83	[0.73-0.95]	0.005	0.22	[0.19-0.25]	<0.001	0.87	[0.72-1.04]	0.130
South East	0.80	[0.70-0.92]	0.001	1.48	[1.25-1.76]	<0.001	0.30	[0.25-0.37]	<0.001
South South	0.62	[0.55-0.71]	<0.001	0.33	[0.28-0.38]	<0.001	0.48	[0.40-0.57]	<0.001
**Place of residence**									
Urban	1.00			1.00			1.00		
Rural	0.76	[0.70-0.82]	<0.001	0.65	[0.59-0.71]	<0.001	0.81	[0.72-0.91]	<0.001
**Women's level of education**									
No formal education	1.00			1.00			1.00		
Primary	1.52	[1.37-1.68]	<0.001	1.48	[1.32-1.66]	<0.001	1.42	[1.21-1.67]	<0.001
Secondary and above	1.76	[1.57-1.97]	<0.001	2.54	[2.24-2.89]	<0.001	1.68	[1.42-1.20]	<0.001
**Women's working status**									
No	1.00			1.00			1.00		
Yes	1.33	[1.15-1.33]	<0.001	1.03	[0.94-1.13]	0.550	1.17	[1.04-1.32]	0.008
**Husband's level of education**									
No formal education	1.00			1.00			1.00		
Primary	1.65	[1.49-1.83]	<0.001	1.27	[1.11-1.43]	<0.001	1.25	[1.05-1.49]	0.013
Secondary and above	1.63	[1.47-1.81]	<0.001	1.61	[1.42-1.81]	<0.001	1.41	[1.19-1.68]	<0.001
**Wealth index**									
Poor	1.00			1.00			1.00		
Middle	1.83	[1.67-2.01]	<0.001	1.80	[1.61-2.00]	<0.001	1.37	[1.17-1.61]	<0.001
Rich	2.24	[2.00-2.49]	<0.001	2.94	[2.60-3.32]	<0.001	1.83	[1.55-2.17]	<0.001
**Religion**									
Traditional/other	1.00			1.00			1.00		
Christianity	1.14	[0.83-1.57]	<0.001	1.63	[1.07-2.46]	0.021	1.24	[0.65-2.39]	0.511
Islam	1.15	[0.83-1.58]	<0.001	1.11	[0.73-1.69]	0.620	0.92	[0.48-1.76]	0.792
**ANC use (at least 4visits)**									
No	na	na	na	1.00			1.00		
Yes	na	na	na	2.16	[1.99-2.34]	<0.001	4.67	[3.95-5.54]	<0.001
**Place of delivery**									
Home	na	na	na			na	1.00		
Health facility	na	na	na			na	2.84	[2.20-2.80]	<0.000

## Discussion

The purpose of this study was to determine the factors influencing the utilization of antenatal care (ANC), health facility delivery and postnatal care among Nigerian women using the 2013 Nigeria DHS data set. Use of ANC as well as institutional delivery remains some of the important strategies in reducing maternal and child morbidity and mortality [33]. Nigeria continued to be one the largest sources of maternal and child mortality worldwide [3, 34] and therefore, investigating the determinants of ANC, institutional use of delivery as well as postnatal care will provide evidence for policy directions and basis for programmatic planning as we approach end of 2015 and begin to plan for the post-2015 development agenda.

### Antenatal care

The results from this study showed that at least 51% of women had at least four ANC visits during their last pregnancies which fall short of the required level of 90% [2]. In comparison with other countries in sub-Saharan Africa, Nigeria is behind seventeen other countries in terms of ANC coverage; these countries include Ghana (78.2%), Benin Republic (58.2%), Liberia (78.1%), Sierra Leon (76%), Lesotho (70.4%) and Zimbabwe (64.8%) [35]. This study identified several factors that indicate strong positive influence on the utilization of ANC services: age of mother, place of residence (rural/urban), mother's and husband’ level of education, working status of the woman, household wealth quintile, health insurance enrollment, religion and woman's decision-making autonomy. The factors identified here as determinants of ANC use are consistent with those identified by Simkhada [36] in their systematic review of literature of the determents of antenatal care utilization in developing countries. In this study, we found that being in the age group of 35 and above consistently increased the odds of utilization of ANC by about over 200%. The influence of maternal age on the use of ANC is unclear and inconsistent; some researchers suggests that women in their thirties are more likely to use ANC services compared to those that are younger as observed in our study. This inconsistent finding could be due to confounding from parity; among older women it could be due to previous unpleasant experience with ANC services (quality, content, derived benefits and satisfaction with received care) or due to previous uneventful pregnancies that women now perceive ANC as unnecessary. Among the younger women (including teenagers) it could be lack of knowledge of the benefits of ANC, or the pregnancy could be unwanted and sought care less [36]. The unexpected findings are that pregnant women in North Central and North East were more likely than those of South West to have ANC even though in both situations (North Central and North East) the odds ratios were not significant. This finding is unexpected since coverage of ANC in South West is one of the highest, at 80% and drawing from relative show-economic advantage of the South West over that of North East and North West it is expected that ANC utilization in South West is better compared to North East and North West [1, 37]. Rural location negatively affected the utilization of ANC by about 53%. Previous studies have documented how urban residence confer some advantages on the use of ANC; for instance studies in Nepal, Ecuador and India reported that residing in urban areas increases the chances of use of ANC services [38–40].

In most developing countries including Nigeria, this finding is not unexpected since there is inequity in the distribution and location of health care facilities in favour of urban areas and therefore women in those urban areas have increased accessibility compared to their rural counterparts. This study further revealed that education of the mother and that of her husband are significant predictors of ANC use. Both factors showed a dose-response relation between level of education and likelihood of use of ANC; women with tertiary education were more than four times more likely to use ANC than those with no formal education likewise women whose husbands possessed tertiary level education were about 4.13 times more likely to use ANC than women of husbands with no formal education. Again, this result is similar to those reported by Pallikadavath, Mekonnen and Mekonnen, Nielsen and Kabir [13, 41–43]. Education influences use of health care services synergistically with other covariates such as urban location, employment, health insurance and awareness of benefits of utilization of health services. The more the education, the more likely for the women to live in urban areas where health services are available and education increases the affordability of health services as well as increasing the awareness and knowledge of benefits to be derived from its use which motivate use. Additionally, women who are educated are more likely to have paid employment and to contribute to the household expenditure and consumption and that means more power in decision-making process in household issues including utilization of health services. Household wealth status significantly predicts the use of ANC; women living in rich households are three times more likely to receive adequate ANC than those in the lowest quintile. This is also in conformity with findings from previous studies [44, 45]; household economic status improves both financial and geographic access to health services. The role of religion appears to be similar between Christianity and Islam. Being either a Christian or Muslim increases the likelihood of attending ANC by similar amount. But belonging to traditional or other forms of religious beliefs significantly decreased the probability of adequate use of ANC. Few studies in the past have documented the positive impact of Islamic religion on use of ANC; these studies include those Pallikadavath and Mekonnen and Mekonnen [41, 42].

Relatively new covariates added to the model to assess their influences on maternal health service are health insurance and woman's autonomy on use of ANC. Based on 2013 Nigeria DHS, about 1.8% of women are enrolled into health insurance scheme in Nigeria and the result indicates that it increases utilization among those enrolled compared to those who are not. In Ghana, similar results were reported on the positive effect of health insurance on the utilization of maternal health care services [46–48]. The role of women autonomy as it influences use of maternal and child services have been studied by several researchers in the past; of note is that by Bloom [5]. Story and Burgard reported that joint decision between the wife and husband predicts positive use antenatal care and skilled delivery compared to husband-only [49]. Our adjusted model for use of ANC revealed an increased chance of ANC by the woman-husband pair but not statistically significant. As for facility delivery, woman-alone influences health facility delivery more than woman-husband pair; that is if both the wife and husband are jointly making decision about place of delivery then the woman has a chance of 30% to deliver in a health facility compared to when the woman alone is making the decision which is 36%.

### Institutional delivery

It is estimated that every year about 60 million births occur outside the health facility at home, of which 52million of these births are not attended by skilled medical personnel [50]. Access to skilled care at birth is one the strategies that can reduce both maternal and child mortality; however this access is lowest in sub-Saharan Africa and South Asia and the rate of annual increase in access is mere 0.2% which is not enough to achieve the target of 90% by 2015 [51, 52]. Current body of knowledge has identified the following factors as significant determinants of facility delivery in sub-Saharan Africa: maternal education, parity, place of residence (rural/urban), household wealth, distance to nearest health facility and number of ANC visits [53]. Our adjusted model has identified the following factors to be consistently significant in predicting facility delivery: geopolitical zone of residence, place of residence (rural/urban), maternal education, husband's level of education, household wealth, parity, use of ANC, health insurance coverage and type of decision maker about woman's health spending. Maternal education appears to be most powerful predictor of facility delivery, it is significant in our two models and it also shows a typical dose-response characteristics of with increasing level of education there is corresponding increased chance of facility delivery to the point that women with tertiary level of education are more than two-and-half times more likely to deliver at health facility. Related to maternal education is the influence of husband's level of education which also plays a similar role; increasing level of husband's education increases the use health facility by the woman for delivery. Educated husband are more likely to provide support to their wives to utilized formal health services such as facility delivery [54]. The role of partner's level education on health facility delivery has not been on the spotlight in Nigeria until recently; perhaps the work of Aremu [12] might be among the first few to highlight this important factor. We found that husband's level of education has a positive influence on health facility delivery up to a point where women whose husbands had tertiary level education have about 61% increased probability of facility delivery. Previous researches showed a rural disadvantage in utilization of health services, here also rural residence confers some level of disadvantage on the use of health facility for delivery; those in rural areas were about 35% less likely to use health facility for delivery. The rural disadvantage could be due to access, cost of services, distance and travelling time as well as opportunity cost of leaving place of work to attend health facility and lack of skilled personnel at these health facilities that can lead to poor outcome and poor satisfaction. This finding is not surprising considering the acknowledged spatial distribution of health facilities in low and medium income countries like Nigeria as well as low socioeconomic level of rural people that serves as financial barrier to access health service [55, 56]. Regarding religion, though the odds ratio indicates increased utilization of health facility for delivery, this is not significant. Presence of male medical personnel may deter some Muslim women from accessing health facility for delivery and may use traditional birth attendants for this purpose [56].

Household wealth, parity, enrolment into insurance scheme appeared to be common factors responsible for both use of ANC and facility delivery; and that use of ANC positively predicts use facility delivery. These findings are common in literature where an inverse relationship between parity and use of facility delivery [57, 58]; positive relationship between household wealth index with facility delivery [12, 59, 60] and increased use of facility delivery as a result of health insurance enrolment [12, 61–63]. Our study therefore, further confirms the pattern and nature of the influence of these factors on the utilization of facility delivery in Nigeria. Previous findings relating to utilization of facility delivery to ANC use is that of positive influence of ANC on use of facility delivery. This study was able to identify this positive role played by ANC utilization on facility delivery; a woman who had at least four ANC visits was more than two times more likely to have delivered in health facility than those with zero ANC attendance. Previous studies documented that ANC-related factors that increased chance of facility delivery include the timing first ANC visit, number of ANC visits, attended by a doctor, quality of ANC and being advised to deliver in health facility [21, 31, 53, 54, 58]. Postnatal care appears to be the component of maternal and child care service poorly utilized despite being provided at a critical period for both the survival of mother and baby. Large proportions of maternal and child deaths occur within the first 48hours after delivery, therefore provision of adequate postnatal check-up for both the mother and baby will avert large amounts of maternal and child deaths; an estimated 310,000 newborn lives (or 10-27%) could be saved by postnatal care coverage of 90% [2]. Thus, to prevent maternal and child death, it is recommended that both the mother and newborn baby have at least three postnatal care within seven days of delivery [63]. Our results indicate that approximately one-in-three newborns received postnatal care within two months of delivery. Again, age of mother, geopolitical zone, place of residence, maternal and husband levels of education, wealth level, parity, pregnancy wantedness, ANC attendance, health facility delivery and the position of the decision maker are the uniform predictors of postnatal care. Social advantage as reflected in high wealth index, high maternal and husband's levels of education and urban location are significantly associated with utilization of postnatal care. These social advantages, as pointed out earlier act synergistic to make it easier for the mothers to use postnatal care for their newborns. For instance, woman with tertiary education is more likely exposed to benefits of postnatal care, more likely to have had ANC and delivered in health facility and therefore these might propel her further to access postnatal care. Living in urban towns/cities made her more exposed to facilities providing postnatal care and that she or her husband might be gainfully employed that makes health services affordable in their places of residences. In this type of family, misconceptions about postnatal care is unlikely to be tenable, and other barriers to utilization of health care such as distance to facility, lack of confidence and the need to be attended by female health worker are unlikely to play any major role as has been elucidated by some investigators [64–68].

A finding of note is the relationship between postnatal care and parity; the higher the parity the less likely to receive postnatal care. It is possibly related to maternal experience of child birth to extend that those high parity women do not consider postnatal care worthwhile from experience they gather from previous child birth. This might also explain the reason why higher parity women use facility for delivery as well as ANC less than lower parity as seen in this study. Our study further confirms the influence of ANC and facility delivery on access of postnatal care. ANC provides an opportunity for sending message to the attendees on the benefits of facility delivery and postnatal care. Further, delivery in health facility affords the chance to access postnatal care, even though this is not spontaneous. This relationship has been documented by Titaley [68].

### Strengths and limitations

An important strength of this study is the utilization of a nationally-representative sample of women as respondents. With this approach, national averages are generated for the whole country. However, as can be seen, there are a lot of regional variations the estimates generated; though this could be provide region-based policy direction and programming. The study is further limited by the fact that is based on recall of events that could have happened some five years earlier. The study design, a cross-sectional nature made it only possible to make some probabilistic conclusions; from cross-sectional studies allow generation of hypotheses only, testing such hypotheses and drawing causal inferences requires experimental study designs.

## Conclusion

This study corroborates previous research investigations on the potential roles of maternal and husband's level of education, place of residence (urban/rural), parity and wealth level in predicting utilization of MCH services. These factors consistently predicted utilization of all the three indicators of maternal and child health services, that is ANC, institutional delivery and postnatal care. ANC positive influences place of delivery as well as postnatal care while place of delivery influences postnatal care. Non-uniform predictors of these MCH indicators are maternal age, geopolitical zone, employment status of mother, pregnancy wantedness, and health insurance coverage and decision maker.
